# A new chapter for JACMP: vision, article types, and new initiatives

**DOI:** 10.1002/acm2.70536

**Published:** 2026-03-10

**Authors:** Yi Rong, Ingrid Reiser

**Affiliations:** ^1^ Department of Radiation Oncology Mayo Clinic Phoenix Arizona USA; ^2^ Department of Radiology University of Chicago Chicago Illinois USA

## 1 INTRODUCTION

The Journal of Applied Clinical Medical Physics (JACMP) was established with the support of the American Association of Physicists in Medicine (AAPM) as an international Gold Open Access journal dedicated to the clinical medical physics community. With 25 years of growth, the journal has evolved into a highly impactful and international journal, with more than 1.3 million downloads annually. It is our great pleasure to present this first editorial after assuming the roles as the Editors‐in‐Chief of the journal starting in 2026. The journal now stands at a pivotal point in its evolution, while the medical physics profession is advancing rapidly in technological innovation and clinical implementation. This article outlines our vision, new initiatives, expansion in article types and categories, as well as the journal's future development.

## 2 THE VISION OF THE JOURNAL

The vision of our new editorial team is to further strengthen the journal as a global leader for the clinical medical physics profession. Building on the foundation established by the previous editorial team, we will continue to prioritize content that is directly relevant to clinical research, patient care, and day‐to‐day practice, including the implementation of clinical protocols, methodologies, guidelines, consensus statements, practical case studies, educational tools, regulations and professional topics. We envision JACMP as a global resource that busy clinical physicists can readily learn from, adopt, and apply in their own clinics. In addition, we aim to create a meaningful platform for supporting and promoting early‐career professionals, medical physics trainees, and other healthcare contributors.

## 3 THE SCOPE AND OVERVIEW OF THE JOURNAL

**FIGURE 1:**
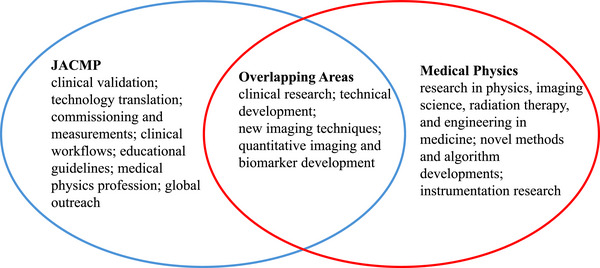
Journal scopes for JACMP and medical physics.

Multiple actions have been initiated during the first two months of our new editorial team. These actions focus on three areas: journal operations, content development, and the enhancement of global visibility. From an operational perspective, our actions include clarifying the journal's scope, expanding the international editorial team members, shortening the peer review timeline, and improving clarity on guidelines for authors and reviewers. JACMP publishes clinical research, technical innovation and developments, educational guidelines, global outreach, and professional perspectives that advance the practice of medical physics in radiation oncology, diagnostic imaging, interventional radiology, nuclear medicine, health physics, and related disciplines. In contrast to the Medical Physics journal, which places greater emphasis on fundamental and methodological research, JACMP focuses on the clinical translation, validation, and implementation of new technologies, products, and technical innovations. As a journal founded for medical physicists and the profession, JACMP also serves as a platform for articles covering professional practice, education, and global engagement. Figure 1 summarizes the distinct focuses and scopes of the two journals. While there is a broad range of topics that appropriately fall within the scope of both journals, JACMP is primarily clinically and professionally oriented, whereas Medical Physics is strongly centered on novelty‐driven research. We also would like to acknowledge the support from the Editors‐in‐Chief of the Medical Physics Journal, Dr John M. Boone and Dr Stanley H. Benedict, who had reviewed and approved the figure.

## 4 THE NEW EDITORIAL TEAM, ROLES, AND RESPONSIBILITIES

The new editorial team responsible for the journal operation is composed of one editor‐in‐chief (EIC), one deputy editor‐in‐chief (DEIC), and six section editors (SE). These carefully selected and highly devoted professionals are diverse in expertise, knowledge, and geographical location. This editorial leadership team ensures efficient peer‐review workflows, clearer and consistent standards, publication quality and scientific rigor. Detailed biographies and keywords for each editor are provided in the Supporting Information. We also have more than 100 dedicated associate editors (AE) on the board. The AE board covers a wide range of medical physics specialties and is fully committed to providing fair and rigorous editorial judgment for each submission. Needless to say, the journal relies heavily on our volunteer reviewers, who graciously contribute their time and expertise to ensure high‐quality publications for our journal. Additionally, our department editor team is constructed for specific article types that need commissioning or the areas in which the journal has potential for development. The Department Editors for Review Articles, Dr Xun Jia, Dr Xuanfeng Ding, and Dr Kai Yang, are responsible for reviewing proposal submissions and inviting subject experts to contribute to our three categories: Topical Review, Best Practice Consensus, and Vision Articles (see more details in the Section 7). The Parallel‐Oppose Debate has been a flagship initiative that brings readers' attention and awareness to controversial topics in the field. These articles are now carried out more broadly with our two new Debate Department Editors, Dr Dongxu Wang and Dr David W. Jordan. A new initiative titled Global Spotlight Editorial is co‐moderated by Dr Nataliya Kovalchuk and me. This series features interviews with distinguished JACMP contributors, including authors, reviewers, and editors, covering a range of topics in clinical medical physics. Our goal is to present international perspectives and highlight how experts from different countries approach similar challenges in distinct ways, shaped by their training, culture, practice environment, and resources. Dr Jay W. Burmeister and Dr Ashley Cetnar oversee articles submitted to the education categories. Dr Rebecca Milman and Dr Joann I. Prisciandaro oversee AAPM reports and documents. Dr James L. Robar and Dr Jinzhong Yang are Department Editors for Industry Innovation and Grand Challenge. Dr Minsong Cao and Dr Michelle M. Svatos focus on reviewing and inviting patient‐centered studies and physics‐focused data interpretation studies for clinical trials. In an attempt to promote our journal articles on social media, Dr Amy S. Yu has revived the video abstract program for monthly selected Notable Articles. To enhance global visibility, our editors have also been actively engaging social media to introduce new members, increase transparency in the selection processes, and announce our new initiatives for the journal. Additional new initiatives for expanding the journal's overall impact are underway, in collaboration with Department Editors. We will share more information in our next editorial about them and their focus areas. We will also elaborate more on the roles, responsibilities, and strong background of our Department Editors in the next editorial publication.

## 5 JOURNAL OPERATION AND PEER‐REVIEW PROCESS

The new editorial team started to manage journal submissions towards the end of the year 2025. Our new team aims to establish a virtuous cycle for the journal, which includes providing a clear scope definition for authors, encouraging high‐quality manuscript submissions, ensuring efficient and rigorous peer‐review, and eventually sustaining journal quality and overall global impact. The first step to achieve such a virtuous cycle is to improve the author submission experience and reduce peer‐review duration. We have seen great improvement in the efficiency of the peer‐review process. Based on Wiley Insight statistics, the average time from manuscript submission to first decision at JACMP has seen a substantial decrease, with further reductions anticipated in the coming months, thanks to the collective efforts of the entire editorial team and the reviewers. Three key factors have contributed to these improvements. First, during the initial screening stage, with a much larger editorial team allocated for the journal, the EIC, DEIC, and SE are able to carefully review every submission prior to initiating peer review, allowing early identification of out‐of‐scope manuscripts or those that do not meet publication standards. Second, Associate Editors play a critical role in promptly assessing the scientific merit of submissions and working closely with Section Editors to identify manuscripts with low publication potential. This way, reviewer effort is focused on high‐quality studies. Third, our reviewers provide careful and constructive review comments for those submissions having undergone the peer‐review process, which help authors improve their manuscripts toward acceptance for publication.

## 6 GUIDELINES FOR AUTHORS AND REVIEWERS

Clear guidelines for authors and reviewers are essential for ensuring an uninterrupted submission and peer‐review process. Updated guidelines will be posted on the JACMP.org website after going through the publisher typesetting and processing. We encourage authors to carefully read through those guidelines before manuscript submission. In the era of increasing use of large language models (LLMs) in the academic sector, one important aspect for both authors and reviewers is to understand the journal policy regarding the use of LLMs. JACMP strictly follows the COPE guideline for publication ethics https://publicationethics.org/guidance/cope‐position/authorship‐and‐ai‐tools.

For authors: “Authors who use AI tools in the writing of a manuscript, production of images or graphical elements of the paper, or in the collection and analysis of data, must be transparent in disclosing in the Materials and Methods (or similar section) of the paper how the AI tool was used and which tool was used. Authors are fully responsible for the content of their manuscript, even those parts produced by an AI tool, and are thus liable for any breach of publication ethics.”

For reviewers: “Reviewers, including Associate Editors and Referees, are strictly prohibited from entering any manuscript content into LLMs such as ChatGPT, Gemini, or Claude. Manuscripts under review are confidential and may not be shared with external systems or used to generate, draft, or substantively shape reviewer reports. LLMs may be used only for general language polishing of reviewer‐authored text that contains no manuscript content or identifiable details. Use of LLMs in violation of these principles breaches AAPM Journals’ confidentiality and intellectual property requirements.”

## 7 ARTICLE TYPES AND CATEGORIES

**FIGURE 2:**
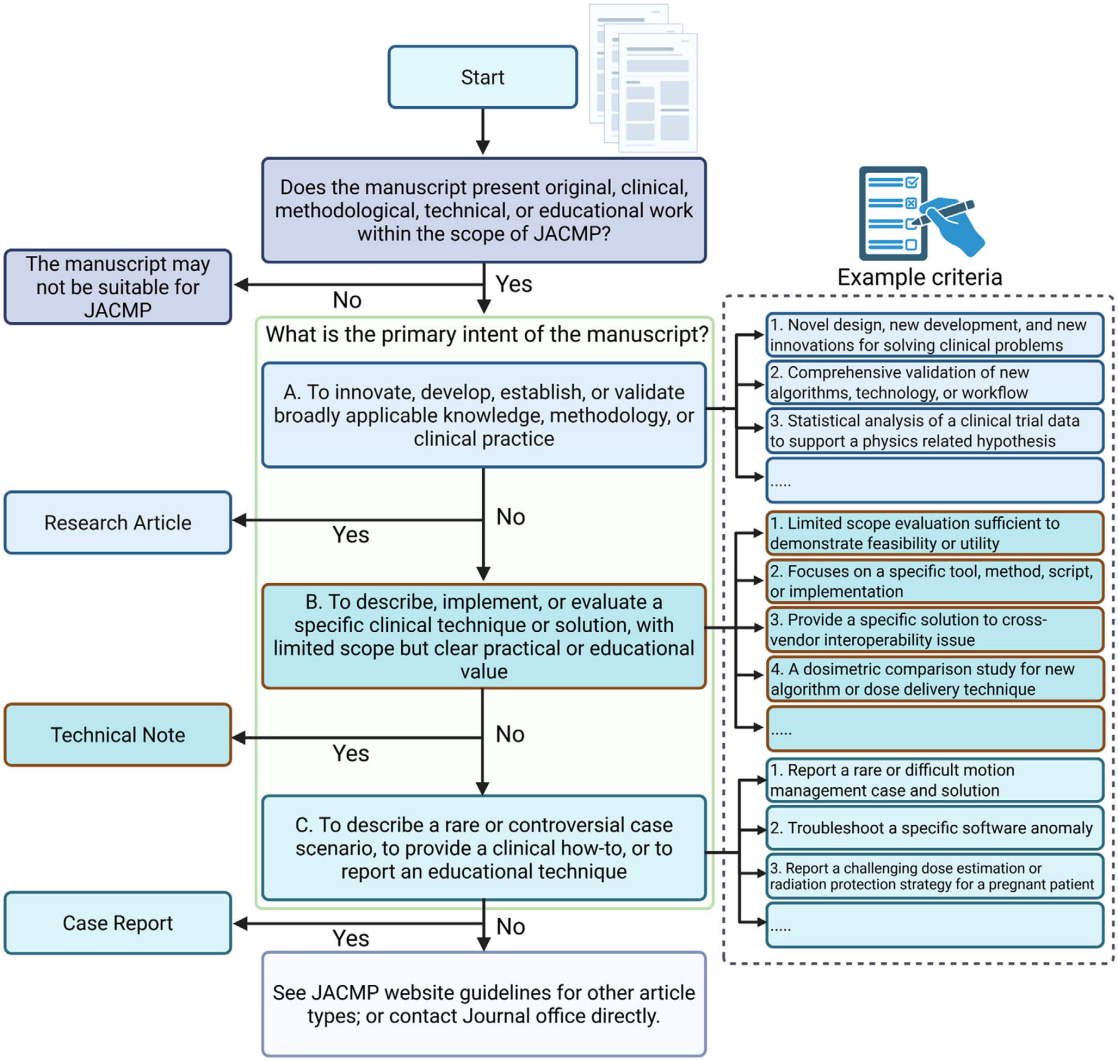
Decision tree flowchart to assist authors in selecting the appropriate submission category among research articles, technical notes, and case reports, including key criteria and examples.

JACMP accepts 12 article types, each with distinct submission or commissioning pathways, peer review requirements, and formatting expectations for abstracts and main text. Brief descriptions of each article type are summarized in Table 1. To further assist authors in selecting the appropriate article type, Figure 2 presents a decision tree flowchart outlining the main considerations, criteria, and examples to help distinguish between the three main submission types, including research article, technical note, and case report. We encourage authors to carefully select the appropriate article type at the time of manuscript submisison. In our next editorial, we will discuss the different peer‐review standards of various article types. Choosing the appropriate article type will faciliate the review process. The editorial team may also adjust the article type to better match the nature of the study when necessary. Furthermore, we expanded the submission categories for three article types: research articles, technical notes, and review articles, as shown in Table 2. These categories will appear as a drop‐down list in the manuscript submission system after authors select the article type. The categories outlined in Table 2 serve three purposes: to help authors select the most appropriate submission type, to encourage submissions across a broad range of topics, and to assist the editorial team in identifying appropriate experts to oversee each submission. These categories are used only during submission and will not appear in the final publication. A brief description is provided for each category to clarify its main focus and distinguish it from the others. A list of recommended limits on total words, figures and tables, as well as the references, is provided in Table 3 for each article type. These limits are intended to encourage conciseness and improve readability for submitted manuscripts. Authors may email the Editor in Chief or elaborate in the cover letter to request an exception if there is sufficient justification for exceeding these limits.

**TABLE 1:** List of 12 JACMP article types, along with publication pathway, structure formatting requirements, peer‐review, and their descriptions.

JACMP article types	Submission or commission	Structured abstract and main text	Peer‐review	Description
Research Article	Submission	Yes	Yes	Original experimental and theoretical research
Technical Note	Submission	Yes	Yes	A concise description of a specific development, procedure or device
Review Article	Hybrid	No	Yes	Topical Review, Best Practice Consensus, and Vision Article
Case Report	Submission	No	Yes	A report on a rare and challenging case, a real‐world problem and solution, or clinical how‐to for a specific topic
AAPM Reports and Documents	AAPM Task Group Submission	No	Yes	A consensus report prepared by an official task group of AAPM
Special Reports	Other organization Submission	No	Yes	A consensus report sponsored by an organization other than the AAPM. **Must include the organization name in the title**
Dataset and Software Articles	Submission	Yes	Yes	A description of open‐access datasets or software with high potential for supporting clinical medical physicists working on related problems. **Must include open‐access links for the dataset or software**
Parallel Opposed Debate	Commission	Yes	No	A concise pro and con position debate on a controversial issue in the discipline, by invitation only.
Editorial	Commission	No	No	Editors’ Editorial presents concise viewpoint offering perspective or commentary pertinent to subject matters in the field of clinical medical physics. Global Spotlight Editorial highlights distinguished medical physicists from diverse regions and practice settings who have made meaningful contributions to JACMP, including reviewers, authors, and editorial leaders. The series features their perspectives on key topics alongside reflections on their professional journeys, by invitation only.
Letter to Editor	Submission	No	No	A brief commentary on a topic or a response to previously published articles, intended to provide constructive criticism, additional insights, or alternative perspectives on the discussed topics
Book Review	Commission	No	No	A concise evaluation and summary of a book's content, significance, and relevance to its intended audience, by invitation only.
Correction	Submission	No	No	A statement by the authors of the original paper that briefly describes any correction(s) resulting from errors or omissions, following templates.

**TABLE 2:** Submission categories for Research Articles, Technical Notes, and Review Articles, with brief descriptions. These categories appear only in the manuscript submission system to help authors select the appropriate article type and to assist the editorial team in identifying suitable experts for review.

Research article categories	Brief description
Radiation Oncology Physics	Research studies for improving radiation therapy on various aspects, including simulation, imaging guidance, planning, and treatment delivery. The studies include, but are not limited to, innovation, developments, commissioning, and evaluations on new methodologies and new systems.
Diagnostic and Interventional Imaging Physics	Research studies on imaging methodology, technologies, optimization, and clinical performance in diagnostic and interventional practices.
Nuclear Medicine and Radiopharmaceuticals	Research studies involving nuclear medicine and radiopharmaceutical imaging, therapy, and dosimetry applications.
Radiation Protection and Safety	Research studies addressing patient, staff, and public radiation protection, safety, and exposure management in medical settings.
Radiation Detectors and Measurements	Development and evaluation of new detectors, measurement methods, and calibration techniques for clinical use.
Education and Mentorship	Structured research studies evaluating training methods, curriculum development, and educational outcomes in medical physics.
Management and Professionalism	Data driven research studies examining clinical operations, workforce, productivity, and professional practice related topics.
Policy and Regulations	Data driven research studies evaluating the implementation and impact of regulatory and policy changes in medical physics and relevant professions.
Clinical Trial Insights	Physics related studies supporting clinical trial design, deployment, and consistency; Clinical trial data analysis in medical physics aspects.
Other Research Topics	Research studies relevant to clinical medical physics not covered in the categories above.

Technical note categories	Brief description
Clinical Pearls	Practical clinical tips or solutions addressing specific technical challenges in daily practice.
Imaging Methods	Implementation or optimization of imaging techniques for clinical application.
Quality Assurance and Incident Learning	Practical approaches or tools for QA and QC processes in imaging or therapy systems.
Dosimetric Studies	Dosimetric evaluations for new commercial algorithms, clinical workflows, or delivery systems.
Implementation and Workflow	Descriptions of system implementation and practical solutions improving clinical workflows or operational efficiency.
Industry Innovations	Early user experiences of new commercial technologies in clinical settings; Vendor white papers on new products.
Education and Training Methods	Practical teaching tools or approaches supporting training and mentoring in medical physics and related professions.
Global Perspectives	Reports describing diverse settings in professions and healthcare environments from a global perspective.
Management and Professional Topics	Diverse topics and viewpoints in management and profession.
New Frontiers	Introducing frontier innovations, novel techniques, and emerging technology that have future benefit to the profession.

Review articles categories	Brief description
Topical Review (Proposal required)	Comprehensive review of current knowledge and developments in a focused area or topic.
Best Practice Consensus (Commissioned)	Invited subject matter experts’ consensus articles providing practical guidance or recommended practices for clinical implementation.
Vision Article (Commissioned)	Invited experts’ opinions and forward‐looking perspectives outlining future directions, challenges, and opportunities in medical physics.

**TABLE 3:** Summary of the word, figure and table, and reference limits for different article types. Word limits account for all elements of the manuscript, including the title page, abstract, main text, figure and table legends, and references.

	Word limit for all manuscript elements	Figure + Table limit	Reference limit
Research Article	7500	10	50
Technical Note	5000	6	20
Review Article	7500	10	100
Case Reports	3000	6	20
Special Reports	7500	10	50
Dataset and Software Article	7500	10	50
Letter to Editor	2000	6	20

## 8 PUBLICATIONS ON ARTIFICIAL INTELLIGENCE AND MACHINE LEARNING

Similar to the Medical Physics journal, JACMP is also observing an increased number of submissions on artificial intelligence (AI) and machine learning (ML) related studies. We encourage authors to follow the checklist for AI/ML applications in Medical Physics (CLAMP)^1^ to ensure rigorous and generalizable research using AI/ML for clinical medical physics. Additional updates and new inititives will be introduced in future editorials.

## Supporting information



Supporting Information
