# Supplementary material for: A new chapter for JACMP: vision, article types, and new initiatives
Source: J Appl Clin Med Phys. 2026 Mar 10;27(3):e70536. doi: 10.1002/acm2.70536 (PMC12975403; doi:10.1002/acm2.70536)
Supplement: Supplementary file 1 — Supporting Information [file ACM2-27-e70536-s001.docx]

Supplementary 1: Detailed biography and key words for each editorial team.


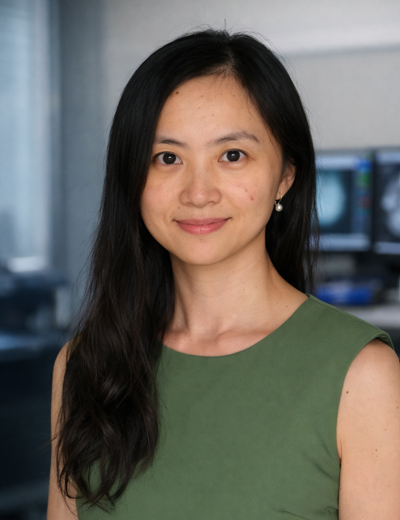
**Editor-in-Chief**: **Dr. Yi Rong** is a Professor of Radiation Oncology and a board-certified therapeutic medical physicist at Mayo Clinic. She received her PhD in Medical Physics from the University of Wisconsin–Madison. Dr. Rong’s work focuses on the clinical translation, validation, and quality management of advanced radiation therapy technologies. She has led or contributed to numerous multi-institutional studies, NRG Oncology clinical trial initiatives, and AAPM task groups. Dr. Rong is a Fellow of the American Association of Physicists in Medicine and has held extensive editorial leadership roles. She is deeply committed to education, mentorship, and advancing the medical physics profession through clinically impactful scholarship and leadership.

**Keywords of expertise:** clinical medical physics, image-guided radiotherapy, simulation-free workflows, artificial intelligence auto-segmentation and auto-planning, cone-beam CT and on-board imaging, motion management, surface guided radiotherapy, brachytherapy, workflow optimization, treatment planning, medical physics education and professional practice; policy and regulations.

Figure S1: Editor-in-Chief: Dr. Yi Rong


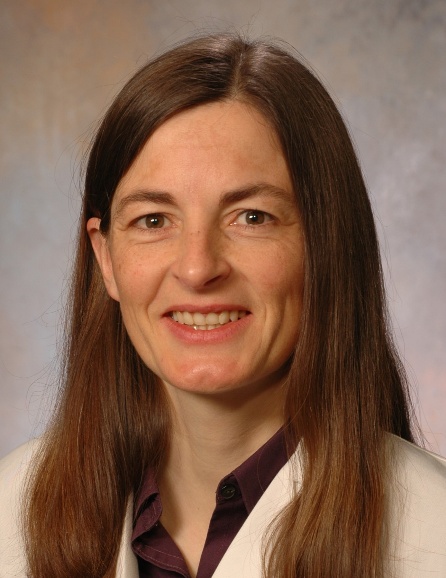
**Deputy Editor-in-Chief: Dr. Ingrid Reiser** is board-certified Diagnostic Medical Physicist and Professor of Radiology at the University of Chicago. She holds a PhD in Physics from Kansas State University. Her research has investigated breast tomosynthesis including image formation, image reconstruction, computer-aided detection and diagnosis, as well as the task-based assessment of imaging systems by use of human and model observers. Her recent research interests include dual energy and spectral CT. Her clinical duties include all aspects of quality control and regulatory compliance of x-ray modalities such as radiography, mammography, fluoroscopy/interventional imaging, and CT, as well as Ultrasound.

**Keywords of expertise:** breast tomosynthesis and mammography, computed tomography, dual-energy and spectral imaging, contrast imaging, CAD, image processing, image reconstruction, digital and physical phantoms, model observers and image perception, quality control and image quality assessment, quantitative and qualitative imaging, radiation dosimetry.

Figure S2: Deputy Editor-in-Chief: Dr. Ingrid Reiser

Figure S2: Deputy Editor-in-Chief: Dr. Ingrid Reiser


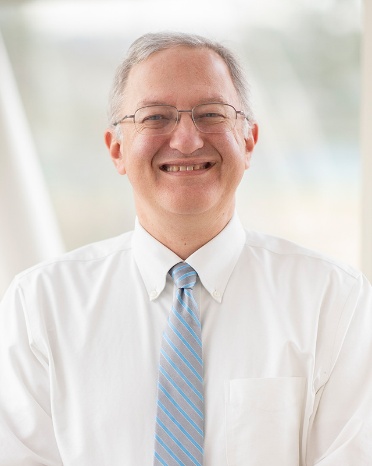
**Section Editor: Dr Trevor Andrews** is an MR physicist and Associate Professor at the Mallinckrodt Institute of Radiology, Washington University School of Medicine in St Louis. He completed his doctoral work UT Health Science Center in San Antonio and a research fellowship at the Vanderbilt University Institute of Imaging Science. While at Philips Healthcare he led development of neurography and synthetic MR products and performed early clinical testing of multi-transmit technology. He serves as Chair of the AAPM MR Subcommittee and Chair of the ABMRS ESC for the MR Safety Expert Exam and was named a Fellow of the AAPM in 2025.

**Keywords of Expertise**: MR imaging; biomarkers; innovations in medical physics education; teaching, learning theory and methods; quality control and image quality assessment; quantitative and qualitative imaging; management and professional topics; policy and regulations topics.

Figure S3: Section Editor: Dr. Trevor Andrews


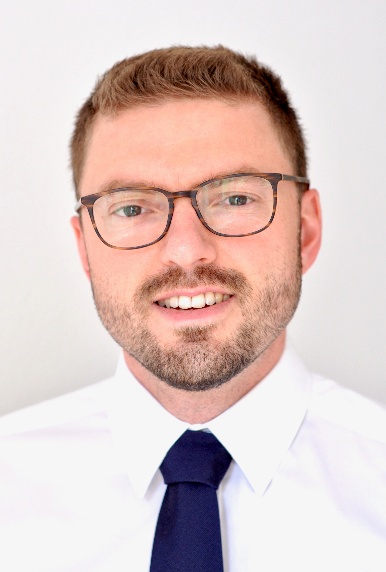


**Section Editor: Dr. Guillaume Landry** is W2 Professor at the Department of Radiation Oncology of the University Hospital of the Ludwig Maximilian University in Munich, Germany, where he holds teaching responsibilities at the Faculty of Medicine. He leads the LMU Adaptive Radiation Therapy Lab, an interdisciplinary research group focusing on advancing image guidance and adaptive strategies in radiotherapy by developing and applying deep learning and artificial intelligence methods to clinical challenges. His work encompasses both online adaptive radiotherapy and motion management, with a strong emphasis on leveraging machine learning to enhance real-time imaging, motion prediction, and treatment adaptation. Dr. Landry’s research activities include contributions to AI-based tumor tracking initiatives and collaborative projects aimed at improving intra-fractional imaging and delivery accuracy within MR-guided radiotherapy workflows.

**Keywords of Expertise**: artificial intelligence, magnetic resonance imaging guided radiotherapy, image guidance, adaptive radiotherapy, spectral computed tomography, cone beam computed tomography, motion management.

Figure S4: Section Editor: Dr. Guillaume Landry

**
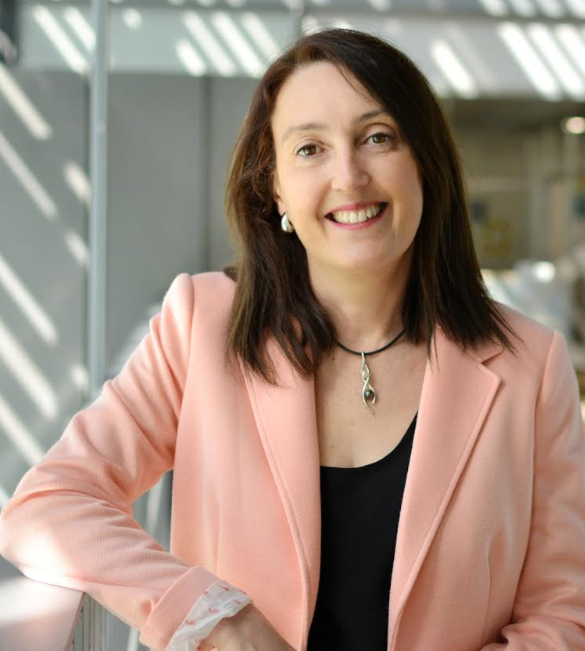
Section Editor: Dr. Yolanda Prezado** is an Oportunius Research Professor supported by the Xunta de Galicia and a board certified medical physicist. She is a Research Director at the French National Centre for Scientific Research, currently on leave, with a dual clinical and research profile in medical physics and radiobiology. She founded the New Approaches in Radiotherapy group and has held senior leadership roles at the European Synchrotron Radiation Facility, CNRS, and Institut Curie. She is the originator of proton minibeam radiotherapy and an ERC Consolidator Grant recipient. She has supervised numerous trainees, led major European scientific committees, and is a frequent keynote speaker.

**Keywords of Expertise**: photon-proton dual specialty, spatially fractionated radiation therapy, mini-beam, flash therapy, small field dosimetry, Monte Carlo simulations, radiobiology, radiation detectors and measurements topics.

Figure S5: Section Editor: Dr. Yolanda Prezado

**
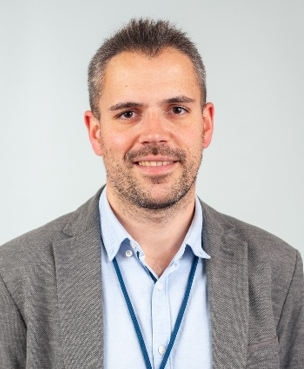
Section Editor: Dr. Alejandro Bertolet** is the Director of Radiopharmaceutical Dosimetry at Massachusetts General Hospital and an Assistant Professor at Harvard Medical School. He conducts NIH-funded research on physical and radiobiological aspects of theranostics, with special focus on both radiopharmaceutical therapy and transarterial radioembolization. He also has extensive expertise in proton and particle therapy, with contributions to dosimetric modeling and radiobiological characterization of charged particle beams for advanced radiation therapy applications.

**Keywords of Expertise:** Radiopharmaceutical therapy dosimetry; targeted alpha therapy; microdosimetry and nanodosimetry; radiobiological modeling of internal emitters; image-based dosimetry (SPECT/CT, PET/CT); Monte Carlo methods in medical physics; transarterial radioembolization (Y-90); Computational modeling and digital twins in radiation therapy; DNA damage modeling and track-structure simulations; radiosensitizing nanoparticles and nanotechnology; proton therapy and dosimetry; particle therapy physics; let based modelling; heavy ion therapy and advanced particle therapy techniques; radiation detectors and measurements topics.

Figure S6: Section Editor: Dr. Alejandro Bertolet


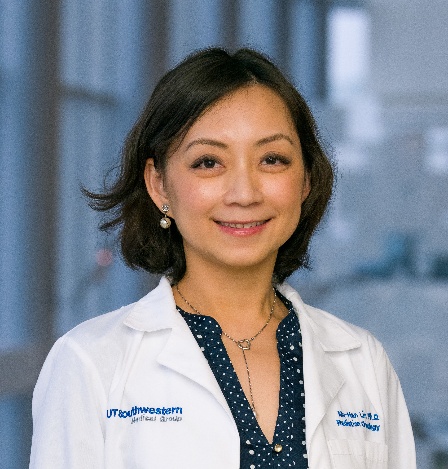
**Section Editor: Dr. Mu-Han Lin** is a Professor and the Senior Director of Clinical Physics at UT Southwestern Medical Center. She oversees clinical physics operations for CBCT-guided and MR-guided adaptive radiotherapy, biology-guided radiotherapy (BgRT), and HDR brachytherapy. Dr. Lin leads multidisciplinary development of advanced treatment-planning and adaptive-therapy techniques, including workflow optimization, automation, AI-assisted contouring, and simulation-omitted planning. Her work focuses on enhancing precision, efficiency, and quality in modern radiotherapy practice. She contributes broadly to AAPM, NRG Oncology, and national working groups and is deeply committed to education, quality, and workforce development.

**Keywords of Expertise:** photon and electron radiation therapy; radiation therapy treatment planning; motion management in radiation therapy; image guided therapy and surgery; MRI guided radiation therapy; artificial intelligence in radiation therapy; immunotherapy combined with radiation therapy; theoretical and computational dosimetry for radiation therapy; supply and demand; staffing; workflow and process management.

Figure S7: Section Editor: Dr. Mu-Han Lin

**
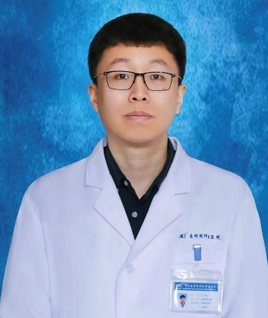
Section Editor: Dr. Kuo Men** serves as the Director of Medical Physicist at Cancer Hospital, Chinese Academy of Medical Sciences. His primary research interests lie in intelligent radiotherapy and medical image processing. To date, he has published more than 60 papers as either the first author or corresponding author. He has been honored with the Young Scientist Award by the International Union of Pure and Applied Physics (IUPAP) and the Best Young Leader Award by the Asia-Oceania Federation of Organizations for Medical Physics (AFOMP).

**Keywords of expertise:** photon and electron radiation therapy; CT technology in radiation therapy; cone beam CT imaging; artificial intelligence in image analysis and radiomics feature extraction; image segmentation; MRI guided radiation therapy; artificial intelligence in radiation therapy.

Figure S8: Section Editor: Dr. Kuo Men
